# Long-Term Warming in Alaska Enlarges the Diazotrophic Community in Deep Soils

**DOI:** 10.1128/mBio.02521-18

**Published:** 2019-02-26

**Authors:** Jiajie Feng, C. Ryan Penton, Zhili He, Joy D. Van Nostrand, Mengting M. Yuan, Liyou Wu, Cong Wang, Yujia Qin, Zhou J. Shi, Xue Guo, Edward A. G. Schuur, Yiqi Luo, Rosvel Bracho, Konstantinos T. Konstantinidis, James R. Cole, James M. Tiedje, Yunfeng Yang, Jizhong Zhou

**Affiliations:** a Institute for Environmental Genomics, University of Oklahoma, Norman, Oklahoma, USA; b Department of Microbiology and Plant Biology, University of Oklahoma, Norman, Oklahoma, USA; c School of Civil Engineering and Environmental Sciences, University of Oklahoma, Norman, Oklahoma, USA; d College of Integrative Sciences and Arts, Arizona State University, Mesa, Arizona, USA; e Center for Fundamental and Applied Microbiomics, The Biodesign Institute, Arizona State University, Tempe, Arizona, USA; f State Key Joint Laboratory of Environment Simulation and Pollution Control, School of Environment, Tsinghua University, Beijing, China; g Center for Ecosystem Science and Society, Northern Arizona University, Flagstaff, Arizona, USA; h School of Forest Resources and Conservation, Department of Biology, University of Florida, Gainesville, Florida, USA; i School of Civil and Environmental Engineering, Georgia Institute of Technology, Atlanta, Georgia, USA; j Center for Microbial Ecology, Michigan State University, East Lansing, Michigan, USA; k Earth and Environmental Sciences, Lawrence Berkeley National Laboratory, Berkeley, California, USA; l School of Biology, Center for Bioinformatics and Computational Genomics, Georgia Institute of Technology, Atlanta, Georgia, USA; Northern Arizona University

**Keywords:** climate warming, diazotrophs, gene sequencing, soil microbiology, tundra

## Abstract

Tundra ecosystems are typically carbon (C) rich but nitrogen (N) limited. Since biological N_2_ fixation is the major source of biologically available N, the soil N_2_-fixing (i.e., diazotrophic) community serves as an essential N supplier to the tundra ecosystem. Recent climate warming has induced deeper permafrost thaw and adversely affected C sequestration, which is modulated by N availability. Therefore, it is crucial to examine the responses of diazotrophic communities to warming across the depths of tundra soils. Herein, we carried out one of the deepest sequencing efforts of nitrogenase gene (*nifH*) to investigate how 5 years of experimental winter warming affects Alaskan soil diazotrophic community composition and abundance spanning both the organic and mineral layers. Although soil depth had a stronger influence on diazotrophic community composition than warming, warming significantly (*P < *0.05) enhanced diazotrophic abundance by 86.3% and aboveground plant biomass by 25.2%. Diazotrophic composition in the middle and lower organic layers, detected by *nifH* sequencing and a microarray-based tool (GeoChip), was markedly altered, with an increase of α-diversity. Changes in diazotrophic abundance and composition significantly correlated with soil moisture, soil thaw duration, and plant biomass, as shown by structural equation modeling analyses. Therefore, more abundant diazotrophic communities induced by warming may potentially serve as an important mechanism for supplementing biologically available N in this tundra ecosystem.

## Abstract (precis)

With the likelihood that changes in global climate will adversely affect the soil C reservoir in the northern circumpolar permafrost zone, an understanding of the potential role of diazotrophic communities in enhancing biological N_2_ fixation, which constrains both plant production and microbial decomposition in tundra soils, is important in elucidating the responses of soil microbial communities to global climate change. A recent study showed that the composition of the diazotrophic community in a tundra soil exhibited no change under a short-term (1.5-year) winter warming experiment. However, it remains crucial to examine whether the lack of diazotrophic community responses to warming is persistent over a longer time period as a possibly important mechanism in stabilizing tundra soil C. Through a detailed characterization of the effects of winter warming on diazotrophic communities, we showed that a long-term (5-year) winter warming substantially enhanced diazotrophic abundance and altered community composition, though soil depth had a stronger influence on diazotrophic community composition than warming. These changes were best explained by changes in soil moisture, soil thaw duration, and plant biomass. These results provide crucial insights into the potential factors that may impact future C and N availability in tundra regions.

## INTRODUCTION

The northern circumpolar permafrost zone contains approximately 1,672 Pg of C, accounting for nearly half of the global soil C storage (1, 2). As the extent of permafrost thaw increases due to global warming, this large soil C reservoir has become increasingly vulnerable to microbial decomposition (2), resulting in a positive feedback to greenhouse gas emissions (3). However, the responses of tundra ecosystems to climate warming vary across ecosystem types and the duration of field experiments, which leads to uncertainty in predicting future C storage. For example, soils of the Alaskan tundra near Eight Mile Lake (EML) have been documented as a C sink during the first 2 years of experimental warming but became a C source after the third year (4–6). In contrast, soil C storage under warming in Alaskan tundra soils near Toolik Lake remained unchanged (7).

The limited soil nitrogen (N) in tundra soils constrains both plant production (8) and microbial decomposition (9), thus strongly affecting the net response of tundra ecosystems to climate warming. Plants and soil microbes compete for essentially the same soil N pool, since they utilize similar N sources (e.g., amino acids, NH_4_^+^, and NO_3_^−^) (10). This is true for tundra ecosystems as well (11), wherein factors such as the spatiotemporal dynamics of N components, roots, and microbes collectively determine the fate of soil N (12). Enlarging the available N pool has a significantly positive impact on tundra plant growth (13). However, the impact of N addition on soil microbes is highly dissimilar between tundra and other ecosystems. For example, addition of N fertilizer inhibited microbial respiration and biomass in forest and grassland soils (14) but enhanced microbial decomposition rates in tundra soils due to the alleviation of N limitation (15). Consequently, the concomitant increase in tundra plant productivity may or may not offset C losses owing to accelerated decomposition associated with a larger soil N pool, which in turn provides an important feedback to global warming.

In addition to the deposit of plant litter N, free-living and plant-associated N_2_ fixation is a major biological N source for the N-limited tundra (14), which is regulated by plant-diazotroph symbiotic interaction (16) and abiotic factors such as temperature and moisture (17). Although plant-associated N_2_ fixers (i.e., diazotrophs) exhibit higher activities than the bulk soil and thus supply more N to plants (18), N fixed by free-living soil diazotrophs is also crucial to the productivity of the ecosystem. Microbial *nifH* genes encode an ATP-hydrolyzing subunit of the nitrogenase complex necessary for biological N_2_ fixation. These genes can serve as a proxy to assess the composition of microbial diazotrophic communities on the basis of *nifH* gene sequences. *NifH* genes have also been used to estimate N_2_ fixing rates based on significant correlations between *nifH* gene abundances and N_2_ fixing rates (19–21). However, these correlational observations do not necessarily suggest that *nifH* gene abundances always correlate with N_2_ fixation rates, since soil edaphic factors and nutrient availability likely influence the strength of these correlations. It was recently shown that long-term (10-year) warming significantly increased *nifH* gene richness and evenness in Oklahoma tallgrass prairie bulk soils (0- to 15-cm depth), suggesting that diazotrophic communities were shaped by warming (22). However, the composition of the diazotrophic community in a tundra soil near EML, Alaska, exhibited no change after short-term (1.5-year) winter warming (5). Therefore, it is important to examine whether the lack of diazotrophic community responses to warming is persistent over a longer time period, a possibly important mechanism in determining tundra soil C stability.

Using deep sequencing of *nifH* gene amplicons, quantitative PCR (qPCR), and GeoChip 5.0 technologies, we launched an integrated study to examine diazotrophs across four depths of a tundra soil at the EML study site, where soils were subjected to a 5-year winter (October to April) warming treatment (4). Longer warming led to a deeper thaw depth and greater soil moisture (1, 6), which in turn affected diazotrophic abundance (23). Therefore, we hypothesize that the longer 5-year warming would significantly increase soil diazotrophic community abundance by stimulating microbial growth and stimulate plant primary production by supplementing biologically available N.

## RESULTS

### Environmental factors.

Warming significantly increased winter soil temperatures throughout all of the four depths, but the effect was weaker for the deeper soils (increase of 0.76 ± 0.22°C in the upper organic layer, *P = *0.026; 0.60 ± 0.11°C in the middle organic layer, *P = *0.005; 0.49 ± 0.11°C in the lower organic layer, *P = *0.008; 0.44 ± 0.12°C in the upper mineral layer, *P = *0.018) (see Table S1 in the supplemental material). The effect of warming lingered into the growing season in deeper soils, evidenced by temperature increases in the upper mineral layer (1.11 ± 0.33°C, *P = *0.026) and the lower organic layer (1.02 ± 0.30°C, *P = *0.029) compared to those in shallower soils (0.40 ± 0.37°C in the middle organic layer and 0.02 ± 0.43°C in the upper organic layer, *P > *0.050). Warming significantly increased the duration of annual soil thaw in all layers, where differences between the warming and control plots were much longer in the upper mineral layer (33.8 ± 6.8 days, *P = *0.006) than in other layers (7.3 ± 1.4 days in the upper organic layer, *P = *0.005; 8.2 ± 3.6 days in the middle organic layer, *P = *0.096; 8.0 ± 2.5 days in the lower organic layer, *P = *0.031) (Table S1 shows the absolute lengths of the durations). Warming also significantly increased soil thaw depth from 18.3 ± 0.4 cm to 23.0 ± 1.5 cm (*P = *0.011) and aboveground plant biomass from 1,617 ± 23 g/m^2^ to 2,025 ± 59 g/m^2^ (*P = *0.035) (Table S1). Other environmental factors remained similar between warming and control plots.

10.1128/mBio.02521-18.6 TABLE S1 Environmental factors. Download Table S1, DOCX file, 0.03 MB. Copyright © 2019 Feng et al. 2019 Feng et al. This content is distributed under the terms of the Creative Commons Attribution 4.0 International license.

Soil depth played an important role in determining many soil factors (Table S1). Soil temperature increased with depth in the winter, while it decreased with depth during the growing season. Soil thaw durations decreased from 146 days/year to 62 days/year with increasing depth. Total C content, measured in dry soil, decreased from 43.1 ± 0.2% to 16.0 ± 2.4% with depth as did total N content, decreasing from 1.24 ± 0.04% to 0.55 ± 0.10% in the deeper soils. Soil bulk density increased from 0.086 ± 0.006 g · cm^−3^ to 0.241 ± 0.024 g · cm^−3^ with depth in the organic layer and was substantially higher in the upper mineral layer (0.965 ± 0.132 g · cm^−3^).

### Total abundance of *nifH* genes.

**FIG 1:**
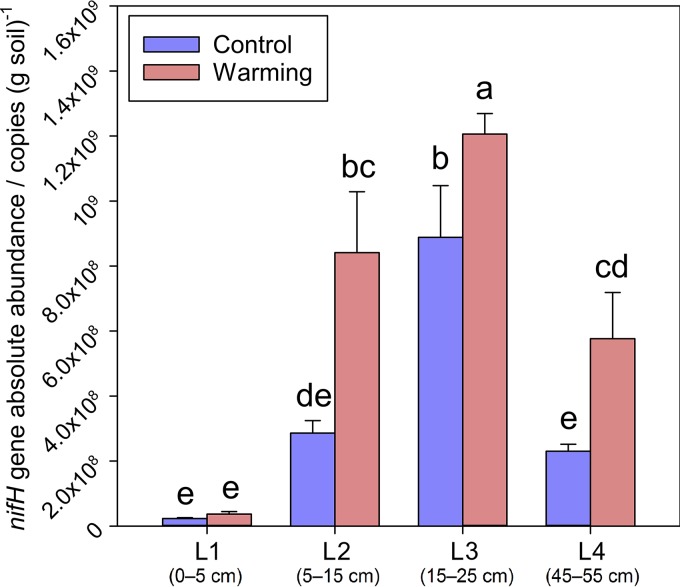
Absolute abundance of *nifH* genes determined by qPCR. Lowercase letters (i.e., a, b, bc, cd, de, and e) above the error bars show the results of ANOVA and LSD tests to examine the significant differences. L1, the upper organic layer; L2, the middle organic layer; L3, the lower organic layer; L4, the upper mineral layer.

In the control plots, the abundance of *nifH* genes was 2.3 × 10^7^ copies/g soil in the upper organic layer (qPCR) (Fig. 1) with a significant increase to 2.9 × 10^8^ copies/g soil in the middle organic layer and to 8.9 × 10^8^ copies/g soil in the lower organic layer. In the upper mineral layer, abundances decreased to 2.3 × 10^8^ copies/g soil. These results were supported by qPCR using operational taxonomic unit (OUT)-specific primers, showing that 6 of 11 OTUs exhibited the highest abundance in the lower organic layer (see Fig. S1A).

10.1128/mBio.02521-18.1 FIG S1 (A) Absolute abundance of the top 11 abundant *nifH* OTUs determined by qPCR. The two panes of the figure are different in the scales of *y* axes. Lowercase letters (i.e., a, b, bc, cd, de, and e) above the error bars show the results of ANOVA and LSD tests to examine the significant differences. (B) Average normalized signal intensity of *nifH* genes determined by GeoChip. L1, the upper organic layer; L2, the middle organic layer; L3, the lower organic layer; L4, the upper mineral layer; *, 0.01 < *P* ≤ 0.05; **, 0.001 < *P* ≤ 0.01; ***, *P ≤* 0.001 by ANOVA. Download FIG S1, TIF file, 1.5 MB. Copyright © 2019 Feng et al. 2019 Feng et al. This content is distributed under the terms of the Creative Commons Attribution 4.0 International license.

Warming significantly increased *nifH* gene abundance in all layers except the upper organic layer (Fig. 1). Together, warming increased *nifH* gene abundance by 86.3% (*P < *0.001). A closer examination showed that warming significantly increased individual OTU abundance in 39% of all cases (11 OTUs, 4 layers), while no decrease of individual OTUs was observed across all of 4 soil depths (Fig. S1A). In agreement with qPCR, GeoChip analysis of *nifH* genes also detected significant increases in relative abundances under warming in all layers, with the exception of the upper organic layer (Fig. S1B).

### Community composition of diazotrophic bacteria.

A total of 691,093 raw *nifH* sequences were obtained. After resampling to 5,905 sequences per sample, a total of 4,663 *nifH* OTUs were generated at 95% amino acid similarity. The relative abundance distribution of OTUs showed a long-tail pattern (see Fig. S2), with the top 28 abundant OTUs accounting for 50.4% of the total sequences. Only 196 (4.2%) OTUs closely (≥95% amino acid similarity) matched to cultured taxa, suggesting that our current database coverage of *nifH* genes remains very limited, at least in the tundra soil environment (24). The most abundant OTU was most closely related to Rubrivivax gelatinosus, which accounted for 9.4% of total sequences. The next 4 abundant OTUs accounted for 5.5%, 4.1%, 3.6%, and 3.0% of total sequences.

10.1128/mBio.02521-18.2 FIG S2 The long-tailed pattern of 4,663 *nifH* OTU relative abundance ranking and distribution based on sequencing data. The abundance of the OTUs on the left of the red line accounts for 50.4% of total abundance. Download FIG S2, TIF file, 0.8 MB. Copyright © 2019 Feng et al. 2019 Feng et al. This content is distributed under the terms of the Creative Commons Attribution 4.0 International license.

**FIG 2:**
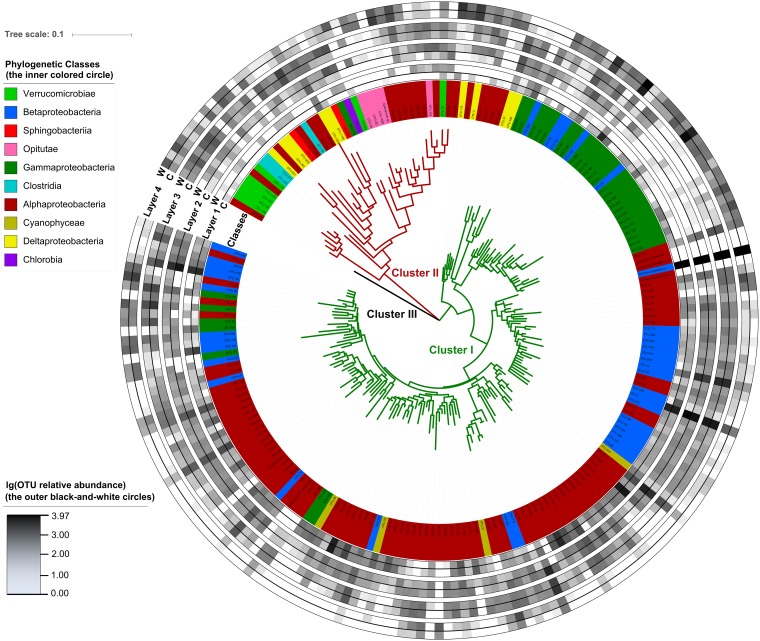
Circular maximum likelihood phylogenetic tree of the top 200 abundant *nifH* OTUs. Tree leaves on the inner circle highlighted by colors show the affiliation of different phylogenetic clades. The outer black-and-white circles show the logarithmic value of OTU relative abundances in percentages in different soil layers and treatments, where tree leaves without abundance are anchored reference taxa. L1, the upper organic layer; L2, the middle organic layer; L3, the lower organic layer; L4, the upper mineral layer; W, warming; C, control.

A neighbor-joining tree for the 200 top abundant *nifH* OTUs shows that closely clustered *nifH* OTUs usually have their closest affiliation to different phylogenetic clades as determined by 16S rRNA genes, implying frequent horizontal gene transfer (HGT) events for these *nifH* genes (Fig. 2) (25). The 3 clusters in the tree match to the 3 previously reported clades of *nifH* genes (26): cluster 1 corresponds to group I, cluster 2 corresponds to group II, and cluster 3 consists of only one OTU of group III. The relative abundances of *nifH* OTUs in cluster 1 showed a decreasing trend with soil depth (23.9%, 41.8%, 31.5%, and 2.7% sequences in the 4 layers), while cluster 2 increased with depth (0.7%, 9.1%, 32.2%, and 58.0% sequences in the 4 layers). Many abundant OTUs were layer specific (e.g., OTU 35, OTU 36, and OTU 130) or treatment specific (e.g., OTU 58 and OTU 753).

The dissimilarities in the compositions of bacterial communities in different soil depths are generally large, with even greater differences than communities that are kilometers apart (27). Consistently, we found that soil depth was a considerably stronger factor than warming on influencing diazotrophic community composition (Table 1). Detrended correspondence analysis (DCA) showed that *nifH* sequences clustered by soil depth rather than by treatment (see Fig. S3). Diazotrophic community composition exhibited significant differences between all layers (*P < *0.001, adonis test) but not between warming and control (see Table S2). Diazotrophic α-diversity indices (richness, Chao1 index, and Shannon index) among any pair of adjacent layers were also significantly different (paired *t* test). These index values increased with depth within the organic layer followed by a decrease in the mineral layer (see Table S3) with abundances illustrating the same pattern.

**TABLE 1:** Adonis test to examine the importance of the effects of warming and depth in shaping diazotrophic community composition (*nifH* gene amplicon sequencing or GeoChip data of *nifH* gene). Abbreviations: Df, degrees of freedom

Factor	df	*nifH* gene sequencing		GeoChip (*nifH*)	
		*R*^2^	*P* value^*a*^	*R*^2^	*P* value^*a*^
Warming	1	0.015	0.217	0.029	***0.056***
Depth	3	0.469	**0.001**	0.386	**0.001**
Warming:depth	3	0.040	0.278	0.056	0.155
Residuals	40	0.476		0.530	

a Bold values indicate *P ≤ *0.05; bold and italic values, 0.05 < *P *< 0.1.

10.1128/mBio.02521-18.3 FIG S3 Detrended correspondence analysis (DCA) of *nifH* gene based on sequencing data. The values in the labels of *x* (11.6%) and *y* (4.3%) axes are percentages that the axis can explain. L1, the upper organic layer; L2, the middle organic layer; L3, the lower organic layer; L4, the upper mineral layer. Download FIG S3, TIF file, 0.7 MB. Copyright © 2019 Feng et al. 2019 Feng et al. This content is distributed under the terms of the Creative Commons Attribution 4.0 International license.

10.1128/mBio.02521-18.7 TABLE S2 Adonis tests of the effects of warming on diazotrophic community composition based from sequencing data and functional structure from GeoChip data for *nifH* genes. Download Table S2, DOCX file, 0.01 MB. Copyright © 2019 Feng et al. 2019 Feng et al. This content is distributed under the terms of the Creative Commons Attribution 4.0 International license.

10.1128/mBio.02521-18.8 TABLE S3 α-Diversity indices, within-treatment β-diversity, and relative abundances (sequence numbers) of *nifH* genes based on sequencing data. Download Table S3, DOCX file, 0.02 MB. Copyright © 2019 Feng et al. 2019 Feng et al. This content is distributed under the terms of the Creative Commons Attribution 4.0 International license.

Warming significantly altered the composition (Table S2) and enhanced the α-diversity (Table S3) of the diazotrophic community in the middle organic layer. Seven of 16 phyla affiliations were significantly altered in sequencing-derived relative abundance by warming (Table S3). The results of GeoChip, which is often more sensitive than sequencing (5), showed a general but weak consistency with the relative abundances derived from the sequencing data (*P < *0.001, *R*^2^ = 0.171) (see Fig. S4A). Warming altered diazotrophic communities in the middle and lower organic layers (Table S2). In agreement with a previous study (28), the value of ln(N_2_-fixer Chao1 index) showed a negative correlation with the reciprocal of absolute soil temperature (*R*^2^ = 0.389, *P < *0.001) (see Fig. S5), suggesting that soil temperature may increase the α-diversity of the diazotrophic community. Warming significantly increased the within-treatment *nifH* β-diversity in the middle and lower organic layers (Table S3), suggesting that warming also increased community dissimilarity within biological replicates.

10.1128/mBio.02521-18.4 FIG S4 (A) Similarity test of diazotrophic community (Bray-Curtis distances are used) between *nifH* sequencing data and GeoChip data. Bray-Curtis distances are used for the similarity test. (B) Similarity test between diazotrophic community based on sequencing data (Bray-Curtis distances are used) and 30 environmental factors (Euclid distances are used). (C) Similarity test between diazotrophic community based on GeoChip data (Bray-Curtis distances are used) and 30 environmental factors (Euclid distances are used). Download FIG S4, TIF file, 1.9 MB. Copyright © 2019 Feng et al. 2019 Feng et al. This content is distributed under the terms of the Creative Commons Attribution 4.0 International license.

10.1128/mBio.02521-18.5 FIG S5 Relationships between diazotrophic richness and temperature. The sequences from all 48 samples were pooled and calculated for theoretical Chao1 value (for individual samples). The natural log values of the Chao1 were used for analyzing the relationships between temperature and diazotrophic richness. The constant *K* (Boltzmann’s constant) equals 8.62 × 10^−5^ electron volt/Kelvin. Download FIG S5, TIF file, 0.7 MB. Copyright © 2019 Feng et al. 2019 Feng et al. This content is distributed under the terms of the Creative Commons Attribution 4.0 International license.

### Drivers shaping diazotrophic community composition.

**FIG 3:**
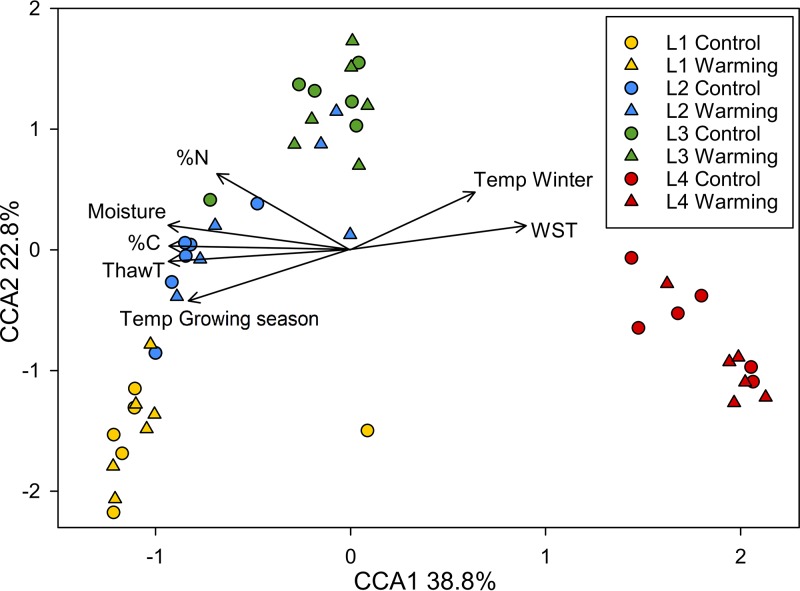
Canonical correspondence analysis (CCA) of *nifH* genes based on sequencing data (circle and triangle symbols) and major environmental factors (arrows). The values in axis 1 and 2 labels are percentages of variations in the diazotrophic community that the axis can explain. L1, the upper organic layer; L2, the middle organic layer; L3, the lower organic layer; L4, the upper mineral layer; %N or %C, soil N or C content; Moisture, soil volumetric water content; ThawT, the duration of soil’s thawed period in growing season; Temp Growing season, soil temperature of growing season; Temp Winter, winter soil temperature; WST, the duration of soil being water saturated during growing season.

Diazotrophic community composition significantly correlated with the measured environmental factors (see Fig. S4B and C). Six top environmental factors (soil thaw duration, growing season temperature, winter temperature, moisture, C content, and N content) explained 88.3% of the variation in the diazotrophic community composition, as determined by a significant (*P < *0.001) canonical correspondence analysis (CCA) model (Fig. 3). Some factors were dependent on specific soil layers. For instance, water saturated time and winter temperature were major factors linking to the microbial community structure within the upper mineral layer. Diazotrophic abundance positively correlated with soil moisture (*P = *0.037), which was tested as driven by the upper mineral layer (see Table S4). For other individual layers other than the upper mineral layer, diazotrophic abundances in the middle organic layer positively correlated with soil thaw duration (*P = *0.053) and plant biomass (*P = *0.015). In addition, diazotrophic abundances in the upper mineral layer positively correlated with soil thaw duration (*P = *0.016).

10.1128/mBio.02521-18.9 TABLE S4 Pearson correlation of diazotrophic abundance between qPCR result and environmental variables. Download Table S4, DOCX file, 0.01 MB. Copyright © 2019 Feng et al. 2019 Feng et al. This content is distributed under the terms of the Creative Commons Attribution 4.0 International license.

**FIG 4:**
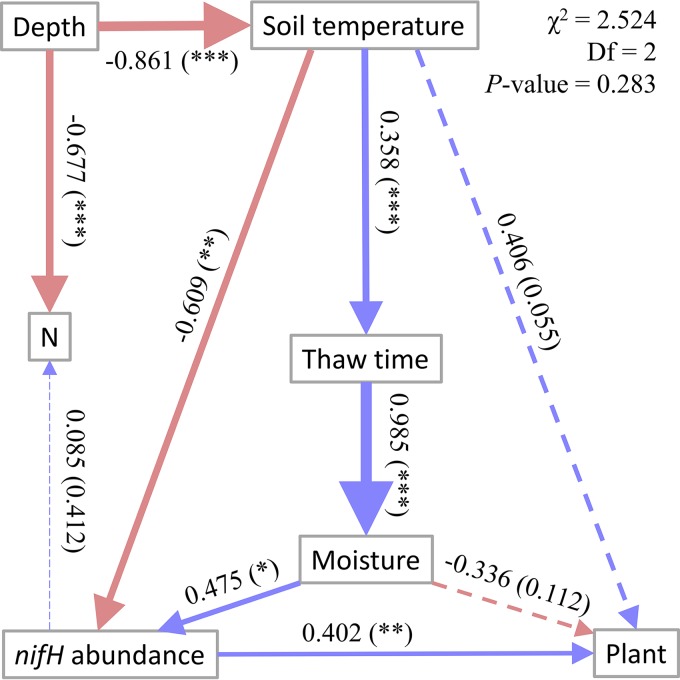
Structural equation modeling (SEM) of *nifH* gene abundance and key environmental factors. Chi-square = 2.524; degrees of freedom = 2; probability level = 0.283. Blue arrows indicate positive relationships, and red arrows indicate negative relationships. Solid lines represent significant correlations, and dashed lines indicate insignificant correlations. Numbers adjacent to arrows are standardized path coefficients (covariation coefficients) proportional to thickness of the lines, with *P* values in the brackets. Significance: *, 0.01 < *P* *≤ *0.05; **, 0.001 < *P ≤ *0.01; ***, *P ≤ *0.001; N, soil N content; Moisture, soil volumetric water content; Thaw time, the duration of soil’s thawed period in growing season; Soil temperature, growing season soil temperature; Plant, aboveground plant biomass.

Structural equation modeling was applied to identify the impacts of environmental factors on diazotrophic abundance (Fig. 4). The interactive model of environmental factors and diazotrophic abundance was well fitted (χ^2^* *=* *2.524, df = 2, *P = *0.283). Diazotrophic abundance positively and significantly correlated to aboveground plant biomass, unveiling a possible interaction between increased diazotrophic abundance and higher plant biomass, and vice versa. Diazotrophic abundance also positively and significantly correlated to soil moisture, which was verified by the result of the Pearson correlation analysis (Table S4).

## DISCUSSION

The experimental warming of tundra soils has been shown to increase soil inorganic N (29). This was previously attributed to N transport from the mineral soils into the upper thawed soil layers when water in the extended thawing layer flows through the deeper soil (30). However, deeper thawing could also export inorganic N from the soil to groundwater and/or surface water (31), thus reducing inorganic N.

N availability has a strong influence in tundra ecosystems, especially through altering plant species composition and enhancing plant growth (32). Since soil warming has been shown to stimulate plant root exudation (33), increased C substrates and decreased soluble N may further stimulate the activity of soil diazotrophs. Long-term soil warming enhanced aboveground plant biomass and foliar N content in Alaskan soil (6), which resulted in enhanced plant competition with soil microbes for N acquisition. As a consequence, the diazotrophic community would be expected to respond to meet the increased N demand when there were favorable environmental conditions. We found that diazotrophic abundance strongly and positively correlated to aboveground plant biomass (structural equation modeling [SEM]) (Fig. 4), which was significantly enhanced by warming (Fig. 1 and Table S1 in the supplemental material). It was shown that warming also affects diazotrophic abundance indirectly via increased soil moisture (23) and water-filled pore space (34) owing to extended thawing, which was consistent with our results (Fig. 4 and Table S4). Whether the tundra ecosystem acts as a C sink or C source depends on both the microbial and plant responses (5), as net ecosystem CO_2_ exchange was reported as increased from −105 to −61 g (CO_2_ C) · m^−2^ (negative values indicate a net C source) by climate warming at the site our samples were taken in May 2013 (35).

The effect of warming on diazotrophic communities varies with soil depth. The temperature sensitivity (*Q*_10_) of soil C decomposition in a temperate forest organic layer was previously shown to be higher than in the mineral layer, since the organic layer contained more C (36). Similarly, we found that warming affected the diazotrophic composition and abundance more substantially in the tundra organic layer than in the mineral layer (Fig. 1 and Table S3). In the upper mineral layer (45 to 55 cm), warming enhanced only diazotrophic abundance (Fig. 1). Transplanting soils to a warmer region has been shown to increase within-treatment β-diversity of microbial taxonomic composition (37). Similarly, warming significantly enhanced the within-treatment diazotrophic β-diversity in the middle and lower organic layers (5 to 25 cm) for both Bray-Curtis and UniFrac distances (Table S3), suggesting that warming enhanced the diversity of the diazotrophic communities. Therefore, it would be challenging to predict diazotrophic composition under a warmer climate due to the higher temporal turnover rates, which is caused by intensified competition or increased niche differentiation as more resources become accessible (38).

Soil depth had a stronger influence on diazotrophic community composition than warming (Table 1 and Fig. 3). Nitrogenase is sensitive to oxygen (39). The arctic tundra soil becomes increasingly saturated by water with depth, leading to oxygen depletion (40). Soil moisture also positively regulates N_2_ fixing rates and diazotrophic community biomass, which could partly be explained by the more anaerobic conditions created by water saturation (23, 41–44). In addition, the lack of photosynthesis in deep soils could further deplete oxygen (45). Therefore, N_2_ fixation rates would be expected to be influenced by depth with concomitant changes in the abundance of the diazotrophs, since correlations between abundance and rate were previously identified (19–21). However, it is important to note that differences in soil environment may influence the strength of these correlations. In addition, the composition of the diazotrophic community may be altered, resulting in different cell-specific N_2_ fixation rates that can impact this correlation. Our results show that diazotrophic abundance within the organic layer (0 to 25 cm) increased with depth, likely due to increasing anaerobiosis and the absence of light in deeper soils. Diazotrophic richness within the organic layer (0 to 25 cm) also increased with depth, which might be attributed to more varied metabolic pathways associated with respiration along the anaerobic continuum. Many plant species living with the stress of anaerobic soil conditions have developed aerenchyma, the enlarged and interconnected intercellular gas spaces in roots and stems to facilitate oxygen utilization (46). Those plant species include Eriophorum vaginatum (47), the most abundant plant species in our site (43.4% of total aboveground plant biomass). It remains unclear how diazotrophic communities, which prefer to anaerobic condition, supply N nutrient to plants. One possibility is through water flow, which provides an explanation for the observation that permafrost thawing increases plant-available N (30). Diazotrophic abundance and richness were low in the mineral layer (45 to 55 cm) (Fig. 1 and Table S3), most likely due to limited C substrates essential for diazotrophic communities (33), given that plant roots reside in the organic layer (47). Within the organic layer, diazotrophic abundance increased along soil depth, but total N content decreased (Fig. 1 and Table S1), which might result from N input of plant litter to surface soil layers.

Extensive sequence reads were obtained in this study, with the majority of the diazotrophic community not taxonomically identified. In the Zehr laboratory’s *nifH* gene database (2014 version), only 16.6% at the genus level and 29.8% at the phylum level of all 41,229 *nifH* gene sequences have taxonomic information. The relative abundances of *nifH* OTUs in cluster I, predominantly composed of aerobic taxa, decreased with soil depth, while those of cluster II, composed of obligate anaerobes, increased with depth (Fig. 2) (26). In addition, closely clustered *nifH* OTUs often belong to different phylogenetic clades, which supports the hypothesis of frequent horizontal gene transfer events for these *nifH* genes (48). The most abundant *nifH* OTU (OTU 7) matched to Rubrivivax gelatinosus, a purple nonsulfur photosynthetic facultative heterotroph capable of growing photosynthetically using CO and N_2_ as its sole C and N sources or anaerobically in the dark (49). The absolute abundance of OTU 7 monotonously increased along soil depth within the organic layer (0 to 25 cm), suggesting that it lives by chemoheterotrophy rather than photosynthesis, and warming significantly enhanced the abundance of OTU 7 in the middle and lower organic layers (5 to 25 cm) (Fig. S1A). Notably, the primer set chosen in this study has been tested in recent studies (24, 50). Although this primer set was evaluated as not capturing most diazotrophs *in silico* (51), the evaluation might be limited in reliability. Indeed, a pair of *in silico* high-performance primers (nifH1/nifH2) generated nonspecific products from soil DNA, which suggested that *in silico* evaluation might not be reliable (51). On the other hand, PolF/PolR (the primer set we used) was evaluated to be reliable in amplifying soil DNA (52).

In conclusion, this study revealed that warming resulted in a more abundant diazotrophic community, though soil depth had a stronger influence on diazotrophic community composition than warming. This result provided valuable insights into the potential factors affecting future C and N availability in tundra regions. Therefore, research is warranted to directly test whether this warming increased diazotrophic community results in increased N_2_ fixation activity and N availability to the ecosystem.

## MATERIALS AND METHODS

### Site descriptions.

The Carbon in Permafrost Experimental Heating Research project (CiPEHR), established in 2008 (4), is located in the EML site on a gentle northeast-facing slope in the northern foothills of the Alaska Range (63°52′59′′N, 149°13′32′′W) (53). Situated within a moist acidic tundra biome, the mean monthly temperature of the site ranges from −16°C in December to +15°C in July. The mean annual temperature was −1.45 ± 0.25°C from 1977 to 2013. The average annual precipitation is 378 mm. The site lies within the southernmost discontinuous permafrost zone, where thawing and thermokarst formation has been occurring over past decades (4). The soil is classified as a gelisol, with a 45- to 65-cm thick organic horizon above a mineral horizon that is a cryoturbated mixture of loess and glacial till. The average active layer depth is approximately 50 cm. Vegetation is dominated by the deciduous shrub Vaccinium uliginosum, and the tussock-forming sedge Eriophorum vaginatum. The tundra soil temperature at the EML site has been monitored since 1985 (54), and ecosystem C fluxes and isotopes have been monitored since 2004 (53).

### Warming experimental design and sample collection.

The winter soil warming treatment at the CiPEHR site was achieved by installing 1.5-m-tall by 8-m-long snow fences between the winter warming and control treatments, perpendicular to the southeasterly dominant winter winds (4). Warming plots were on the leeward side of the snow fences, and the control plots were on the windward side. The snow fences trapped and accumulated an insulating snow layer on the warming plots. Snow was removed before snowmelt in spring (8 to 15 March) to keep the hydrological conditions similar to the control treatment. Snow fences were removed simultaneously to avoid shading the experimental plots during the growing season (May to September). Six snow fences were used for 6 warming-control plot pairs, which were arranged in 3 blocks with fences within a block 5 m apart and the blocks separated by approximately 100 m.

Six soil cores were taken from each treatment in May 2013, after 5 years of winter warming. Soil fractions within the active layer at depths of 0 to 5 cm, 5 to 15 cm, 15 to 25 cm, and 45 to 55 cm were analyzed. The first 3 depths, here referred to as the upper organic layer, the middle organic layer, and the lower organic layer, respectively, belonged to the organic layer and included the depth range in which most plant roots resided. The last depth is referred to as the upper mineral layer.

### Environmental factor monitoring.

Soil moisture was measured using CS616 water content reflectometers (Campbell Scientific, Logan, UT, USA). Soil thaw depths were measured on a weekly basis using a metal thaw depth probe pushed through the unfrozen soil until it hit ice, from which the soil thaw durations were calculated. In the peak growing season, aboveground plant biomass was measured with a nondestructive point-frame method, i.e., using an ∼60- by ∼60-cm point frame with a grid size of 8 by 8 cm to generate 49 intersecting grid points (55). A 1-mm-diameter rod was placed vertically through the grid to touch the plants at each grid point. Plant species identities and tissue types (fruit, stem, flower, or leaf) were recorded. Then, the aboveground biomass was calculated using allometric equations previously developed for this site (53). Soil temperature was measured using type-T thermocouples (Campbell Scientific, Logan, UT, USA). Soil samples were dried at 60°C until reaching a constant weight. Soil samples were then ground and packed into combustion tins for analyses of total C and N contents, using an ECS 4010 elemental analyzer (Costech Analytical Technologies, Valencia, CA, USA).

### Soil DNA extraction.

Soil DNA was extracted via liquid N grinding followed by the PowerMax Soil DNA isolation kit (Mo Bio Laboratories, Inc., Carlsbad, CA, USA) (56). DNA was quantified by Pico green with a FLUOstar Optima fluorescence plate reader (BMG LabTech, Jena, Germany). DNA quality was assessed by a NanoDrop ND-1000 spectrophotometer (Thermo Fisher Scientific, Waltham, MA, USA) based on spectrometry absorbance at wavelengths of 230 nm, 260 nm, and 280 nm. The absorbance ratios of 260/280 nm were larger than 1.8, and the 260/230 nm ratios were around 1.7.

### *nifH* gene amplification and sequence analysis.

Extracted DNA was diluted to 5 ng/µl for amplification. The primers PolF and PolR (TGCGAYCCSAARGCBGACTC and ATSGCCATCATYTCRCCGGA) were used for *nifH* PCR amplification, since they are reliable in amplifying soil DNA (52). Both forward and reverse primers were tagged with Illumina adapter sequence, a primer pad, and a linker sequence. Triplicate PCRs were performed per sample within a reaction volume of 25 µl. PCR products from each sample were separated on a 1.5% agarose gel at 90 V for 50 min. Bands were excised from the gel and then purified with a QIAquick gel extraction kit (Qiagen, Inc., Valencia, CA, USA). Purified DNA was quantified with Pico green, and 100 ng DNA from each reaction was pooled. The pooled DNA was diluted to 2 nM, loaded onto the reagent cartridge, and run on a MiSeq benchtop sequencer (Illumina, Inc., San Diego, CA, USA) at the Institute for Environmental Genomics, University of Oklahoma, according to the manufacturer’s instructions.

Poor quality reads were removed using the Btrim tool (57). Chimeras were removed by Uchime (58) using a manually curated database of *nifH* DNA sequences (59). Frameshifts were screened and corrected by Framebot software (60), again with a manually curated database of NifH protein sequences (59). Remaining sequences were then clustered into OTUs with complete linkage clustering on a Galaxy platform (http://zhoulab5.rccc.ou.edu:8080/) pipeline at the 95% amino acid similarity (61). Phylogenetic trees were constructed and analyzed using PyNAST alignment (v.1.0.0), FastTree (v.1.0.0), and MEGA (v.5.10, BETA2) and visualized using the Interactive Tree of Life (iTOL, v.3.2.2) (62).

### Quantitative PCR.

Quantitative PCR of *nifH* genes was performed with both “universal” (PolF/PolR) primers and 11 pairs of specific primers (see Table S5 in the supplemental material). The specific primers were designed according to DNA sequences of the 11 top abundant (>0.02% of the total abundance) *nifH* genes obtained from our sequencing data. Reactions were performed in 25-µl volumes with the iQ SYBR green Supermix (Bio-Rad Laboratories, Hercules, CA, USA) on a Rotor-Gene 3000 apparatus (Corbett Life Science, Sydney, NSW, Australia). Standards were made from 10-fold series dilutions of plasmids in the TOPO TA Cloning kit (Thermo Fisher Scientific, Waltham, MA, USA) containing 11 *nifH* genes used in the quantitative PCR analyses.

10.1128/mBio.02521-18.10 TABLE S5 Sequences, product sizes, and melting temperatures (*T_m_*) of qPCR primers of the top 11 abundant OTUs. Download Table S5, DOCX file, 0.01 MB. Copyright © 2019 Feng et al. 2019 Feng et al. This content is distributed under the terms of the Creative Commons Attribution 4.0 International license.

### GeoChip 5.0 analyses.

The diazotrophic community was analyzed with a microarray-based tool (GeoChip 5.0), which is the latest version of GeoChip. This microarray contains 161,961 probes belonging to 1,447 gene families, including genes involved in crucial biogeochemical processes (e.g., C, N, P, and S cycling) (63), among which there are 1,331 probes of the *nifH* gene. For each sample, 1 µg of template DNA was labeled with Cy3 dye, purified with the QIAquick purification kit (Qiagen, Germantown, MD, USA) as previously described (64), and hybridized with GeoChip 5.0 M microarrays at 67°C with 10% formamide for 24 h. Subsequently, the microarrays were washed, dried, and scanned on an MS 200 microarray scanner (Roche, South San Francisco, CA, USA). Images were quantified into signal intensities with Agilent’s Data Extraction software. Raw signal intensities were uploaded to the Microarray Data Manager of the Institute for Environmental Genomics at the University of Oklahoma (http://ieg.ou.edu/microarray/) for quality control, normalization, and analysis. We normalized the signal intensity of each spot by mean ratio, removed spots with a <2 signal-to-noise ratio (65), and removed outliers based on standard deviations, as described previously (66).

### Statistical analyses.

Various statistical analyses were performed with the package vegan (v.2.3-2) in R software version 3.2.2 (The R Foundation for Statistical Computing), including diazotrophic α- and β-diversity indices calculated with the package vegan (v.2.3-2) and agricolae, detrended correspondence analysis (DCA) calculated with the package vegan (v.2.3-2) for displaying diazotrophic community structures, nonparametric multivariate analysis of variance (Adonis) calculated with the package vegan (v.2.3-2) for determining differences among diazotrophic community structures, analysis of variance (ANOVA) and *post hoc* Fisher’s least significant difference (LSD) test calculated with the package vegan (v.2.3-2) and agricolae for determining differences among diazotrophic α- and β-diversity indices, canonical correspondence analysis (CCA) calculated with the package vegan (v.2.3-2) for modeling major environmental factors shaping microbial structure, and Pearson correlation analysis calculated with the package Hmisc between environmental factors and diazotrophic abundance. Two-tailed *t* tests were performed using Microsoft Excel 2010 (Microsoft Inc., Seattle, WA, USA). Unless otherwise stated, mean values are given ± standard errors of the means, and *P* values of ≤0.05 are considered statistically significant.

Structural equation modeling (SEM) analysis was performed with the Amos 24.0 software package (Small Waters Corp., Chicago, IL, USA) to establish the structural relationships among the environmental factors and diazotrophic abundance. A chi-square test of model fit was adopted to determine whether the proposed model was supported by the data. Additionally, three other widely used indices of model fit were used, including comparative fit index (CFI), Tucker Lewis index (TLI), and root mean square error of approximation (RMSEA) (67), wherein the good models have a CFI and TLI value of >0.95 and an RMSEA value of <0.05 (68).

### Data availability.

Raw *nifH* amplicon gene sequences are available in NCBI SRA database (https://www.ncbi.nlm.nih.gov/sra) under study no. PRJNA480351. GeoChip raw and normalized signal intensities can be accessed through the URL http://129.15.40.254/NewIEGWebsiteFiles/publications/SupplData/Feng-RawGeoChip-Diazo.txt and http://129.15.40.254/NewIEGWebsiteFiles/publications/SupplData/Feng-NormGeoChip-Diazo.txt.
