# Supplementary material for: Long-Term Warming in Alaska Enlarges the Diazotrophic Community in Deep Soils
Source: mBio. 2019 Feb 26;10(1):e02521-18. doi: 10.1128/mBio.02521-18 (PMC6391920; doi:10.1128/mBio.02521-18)
Supplement: TABLE S1 [file mBio.02521-18-st001.docx]

**TABLE S1** Environmental factors

| Treatments/layers | Winter temperature (^o^C) | Growing season temperature (^o^C) | Soil thaw duration (day per year) | Soil bulk density (g·cm^-3^) | Total N (g/100 g soil) | Total C (g/100 g soil) | Moisture (VWC within 0–20 cm) | Thaw depth (cm) | Plant biomass (g/m^2^) |
| --- | --- | --- | --- | --- | --- | --- | --- | --- | --- |
| Average control L1*^a^* | **-2.3***^b^* | 7.4 | **142.0** | 0.09 | 1.3 | 43.1 | - | - | - |
| Average warming L1 | **-1.6** | 7.4 | **149.3** | 0.09 | 1.2 | 43.0 | - | - | - |
| Average control L2 | **-1.6** | 5.1 | 118.8 | 0.12 | 1.4 | 40.3 | - | - | - |
| Average warming L2 | **-1.0** | 5.5 | 127.0 | 0.10 | 1.4 | 40.0 | - | - | - |
| Average control L3 | **-1.2** | **2.6** | **104.2** | 0.25 | 1.6 | 33.4 | - | - | - |
| Average warming L3 | **-0.7** | **3.6** | **112.2** | 0.23 | 1.6 | 34.7 | - | - | - |
| Average control L4 | **-0.9** | **1.1** | **45.5** | 0.74 | 0.7 | 16.0 | - | - | - |
| Average warming L4 | **-0.4** | **2.2** | **79.3** | 1.19 | 0.5 | 12.4 | - | - | - |
| Average control | -1.5 | 4.1 | 102.6 | 0.3 | 1.3 | 33.2 | 28.1% | **18.3** | **1617** |
| Average warming | -0.9 | 4.7 | 117.0 | 0.4 | 1.2 | 32.5 | 29.2% | **23.0** | **2025** |
| Control p1 L1 | -2.9 | 7.8 | 141.0 | 0.11 | 1.4 | 43.8 | 28.6% | 20.3 | 1729 |
| Control p1 L2 | -1.8 | 5.8 | 111.0 | 0.19 | 1.9 | 38.5 |  |  |  |
| Control p1 L3 | -1.4 | 3.0 | 103.0 | 0.42 | 1.2 | 20.1 |  |  |  |
| Control p1 L4 | -1.2 | 1.5 | 46.0 | 1.07 | 0.1 | 3.6 |  |  |  |
| Warming p1 L1 | -1.5 | 7.0 | 147.0 | 0.06 | 1.3 | 43.0 | 29.7% | 29.6 | 2720 |
| Warming p1 L2 | -0.9 | 5.3 | 135.0 | 0.10 | 1.4 | 39.0 |  |  |  |
| Warming p1 L3 | -0.6 | 3.5 | 117.0 | 0.26 | 1.6 | 31.2 |  |  |  |
| Warming p1 L4 | -0.3 | 1.5 | 99.0 | 1.32 | 0.2 | 5.7 |  |  |  |
| Control p2 L1 | -2.4 | 8.5 | 144.0 | 0.09 | 1.3 | 42.3 | 28.3% | 18.7 | 1633 |
| Control p2 L2 | -1.5 | 5.7 | 125.0 | 0.15 | 1.6 | 39.1 |  |  |  |
| Control p2 L3 | -1.0 | 2.3 | 107.0 | 0.32 | 1.3 | 26.3 |  |  |  |
| Control p2 L4 | -0.9 | 1.4 | 46.0 | 0.50 | 0.7 | 19.8 |  |  |  |
| Warming p2 L1 | -1.8 | 8.1 | 148.0 | 0.08 | 1.0 | 44.1 | 31.5% | 25.5 | 2027 |
| Warming p2 L2 | -1.1 | 5.9 | 128.0 | 0.09 | 1.2 | 41.6 |  |  |  |
| Warming p2 L3 | -0.6 | 4.2 | 116.0 | 0.13 | 1.6 | 37.5 |  |  |  |
| Warming p2 L4 | -0.5 | 3.9 | 94.0 | 1.47 | 0.1 | 2.9 |  |  |  |
| Control p3 L1 | -1.4 | 5.5 | 138.0 | 0.11 | 1.3 | 42.9 | 28.1% | 17.5 | 1508 |
| Control p3 L2 | -1.0 | 3.4 | 117.0 | 0.11 | 1.2 | 40.5 |  |  |  |
| Control p3 L3 | -0.8 | 1.7 | 100.0 | 0.21 | 1.9 | 39.9 |  |  |  |
| Control p3 L4 | -0.4 | 0.3 | 42.0 | 0.95 | 0.5 | 11.8 |  |  |  |
| Warming p3 L1 | -1.4 | 7.4 | 151.0 | 0.10 | 1.5 | 41.7 | 27.8% | 19.7 | 1882 |
| Warming p3 L2 | -0.8 | 5.7 | 121.0 | 0.17 | 1.6 | 39.5 |  |  |  |
| Warming p3 L3 | -0.6 | 3.6 | 107.0 | 0.24 | 1.9 | 34.8 |  |  |  |
| Warming p3 L4 | -0.3 | 2.2 | 75.0 | 0.75 | 0.7 | 18.7 |  |  |  |
| Control p4 L1 | -1.5 | 7.5 | 143.0 | 0.10 | 1.3 | 42.3 | 27.9% | 17.0 | 1344 |
| Control p4 L2 | -1.1 | 5.1 | 125.0 | 0.09 | 1.2 | 41.9 |  |  |  |
| Control p4 L3 | -0.7 | 2.5 | 111.0 | 0.16 | 1.7 | 38.6 |  |  |  |
| Control p4 L4 | -0.6 | 2.0 | 56.0 | 0.40 | 1.4 | 30.9 |  |  |  |
| Warming p4 L1 | -1.3 | 7.5 | 153.0 | 0.09 | 1.2 | 42.3 | 29.4% | 19.9 | 1390 |
| Warming p4 L2 | -0.6 | 5.3 | 121.0 | 0.09 | 1.2 | 40.1 |  |  |  |
| Warming p4 L3 | -0.5 | 3.3 | 107.0 | 0.16 | 1.6 | 38.4 |  |  |  |
| Warming p4 L4 | -0.4 | 2.6 | 68.0 | 0.60 | 0.8 | 21.8 |  |  |  |
| Control p5 L1 | -2.7 | 6.7 | 143.0 | 0.06 | 1.3 | 42.8 | 27.9% | 18.2 | 1820 |
| Control p5 L2 | -1.9 | 4.8 | 118.0 | 0.11 | 1.6 | 39.8 |  |  |  |
| Control p5 L3 | -1.4 | 2.3 | 100.0 | 0.29 | 1.7 | 36.4 |  |  |  |
| Control p5 L4 | -0.9 | 0.3 | 26.0 | 0.26 | 0.9 | 21.5 |  |  |  |
| Warming p5 L1 | -1.4 | 7.4 | 151.0 | 0.10 | 1.1 | 43.1 | 29.0% | 20.4 | 1900 |
| Warming p5 L2 | -1.1 | 5.4 | 132.0 | 0.09 | 1.4 | 40.4 |  |  |  |
| Warming p5 L3 | -0.8 | 3.4 | 114.0 | 0.32 | 1.4 | 30.9 |  |  |  |
| Warming p5 L4 | -0.4 | 1.2 | 71.0 | 1.14 | 0.5 | 12.1 |  |  |  |
| Control p6 L1 | -3.1 | 8.4 | 143.0 | 0.04 | 1.0 | 44.3 | 27.6% | 18.3 | 1671 |
| Control p6 L2 | -2.3 | 5.8 | 117.0 | 0.08 | 1.1 | 42.1 |  |  |  |
| Control p6 L3 | -1.6 | 3.8 | 104.0 | 0.13 | 1.8 | 39.4 |  |  |  |
| Control p6 L4 | -1.1 | 1.1 | 57.0 | 1.29 | 0.3 | 8.4 |  |  |  |
| Warming p6 L1 | -2.0 | 7.1 | 146.0 | 0.08 | 1.3 | 43.6 | 28.0% | 22.7 | 2231 |
| Warming p6 L2 | -1.6 | 5.5 | 125.0 | 0.17 | 1.5 | 36.9 |  |  |  |
| Warming p6 L3 | -1.0 | 3.7 | 112.0 | 0.27 | 1.5 | 35.2 |  |  |  |
| Warming p6 L4 | -0.5 | 1.7 | 69.0 | 1.83 | 0.4 | 13.3 |  |  |  |

*^a^*Abbreviations: L1, the upper organic layer; L2, the middle organic layer; L3, the lower organic layer; L4, the upper mineral layer; p1–p6, plot 1–plot 6; N, nitrogen; C, carbon; VWC, volumetric water content; Thaw depth, soil thaw depth when sampled in May 2013; Plant biomass, peak growing season aboveground plant biomass. This table shows the lengths of soil thaw duration instead of the differences between warming and control.

*^b^*Significance of *P*≤0.05, as determined by two-tailed *t*-test between warming and control, is shown by bold values.
