# Supplementary figures and images for: Long-Term Warming in Alaska Enlarges the Diazotrophic Community in Deep Soils
Source: mBio. 2019 Feb 26;10(1):e02521-18. doi: 10.1128/mBio.02521-18 (PMC6391920; doi:10.1128/mBio.02521-18)

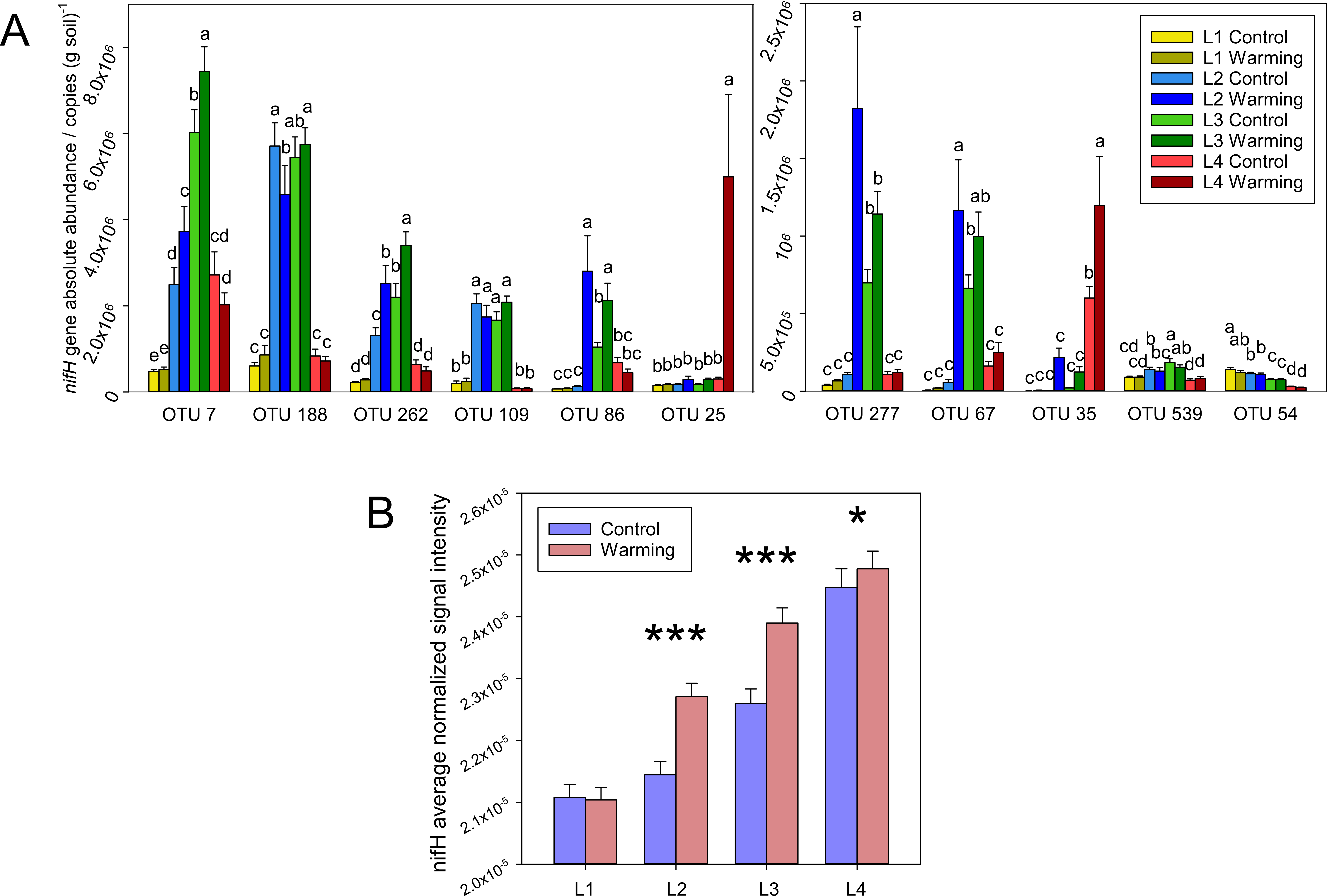

Supplement: FIG S1 [file mBio.02521-18-sf001.tif]

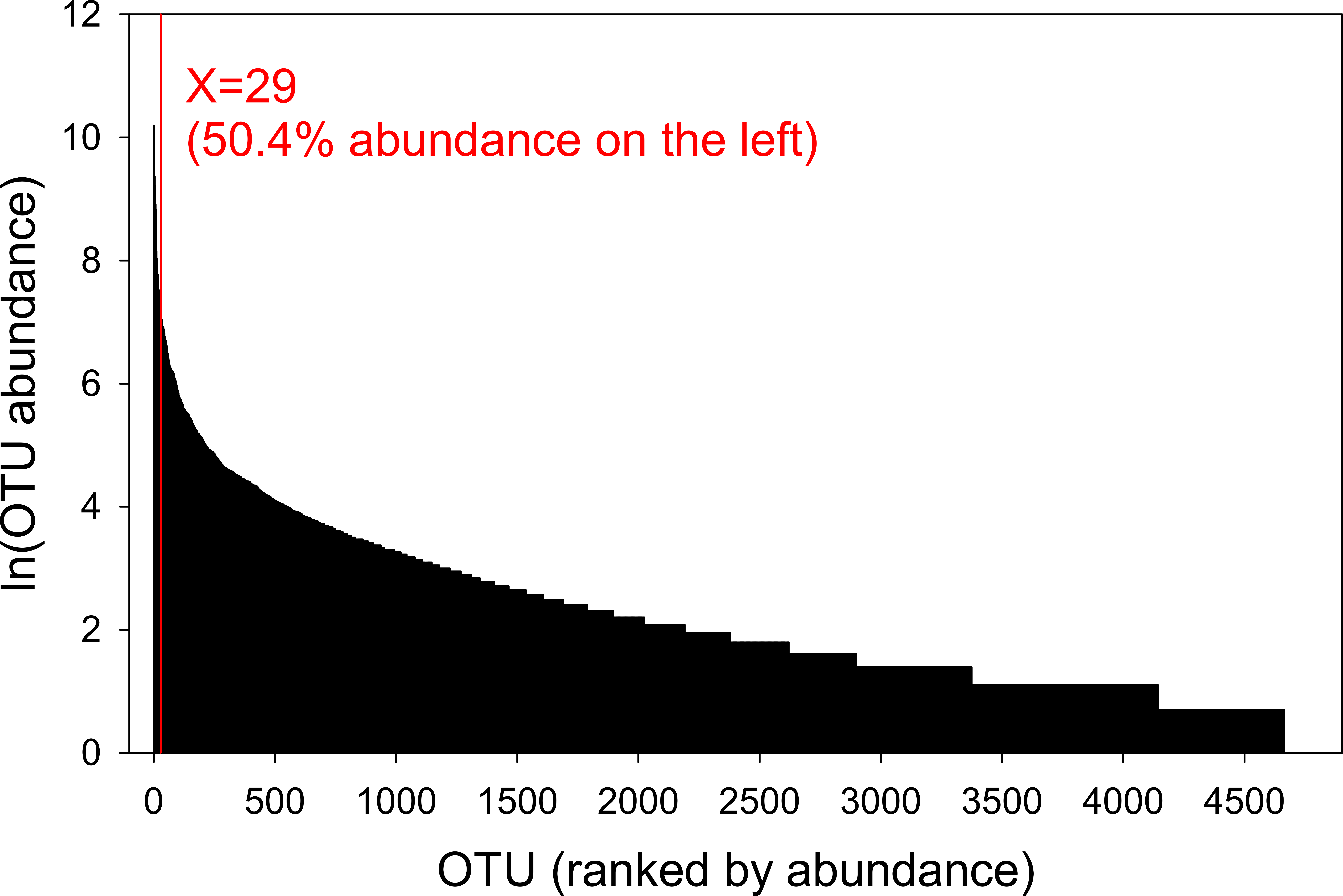

Supplement: FIG S2 [file mBio.02521-18-sf002.tif]

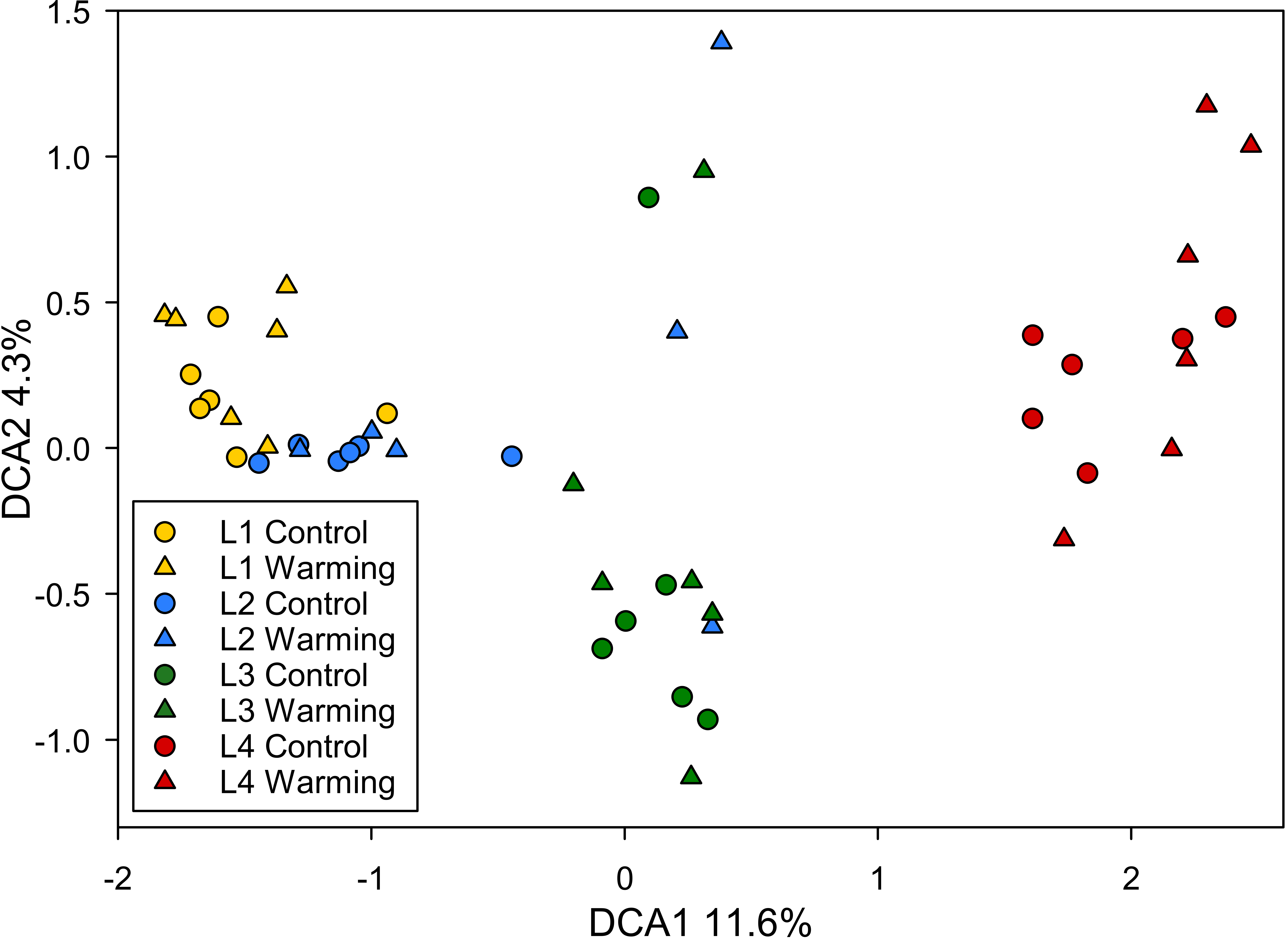

Supplement: FIG S3 [file mBio.02521-18-sf003.tif]

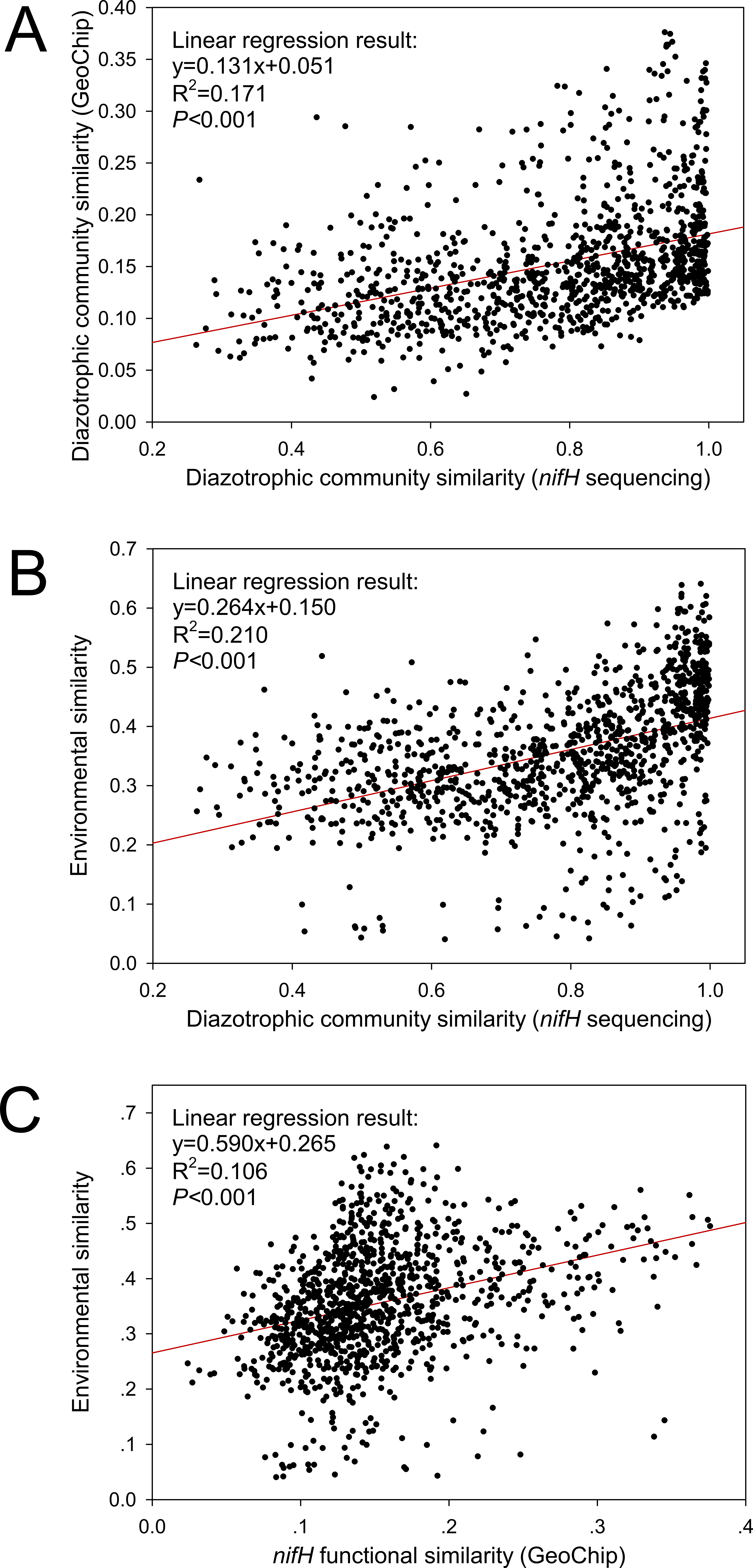

Supplement: FIG S4 [file mBio.02521-18-sf004.tif]

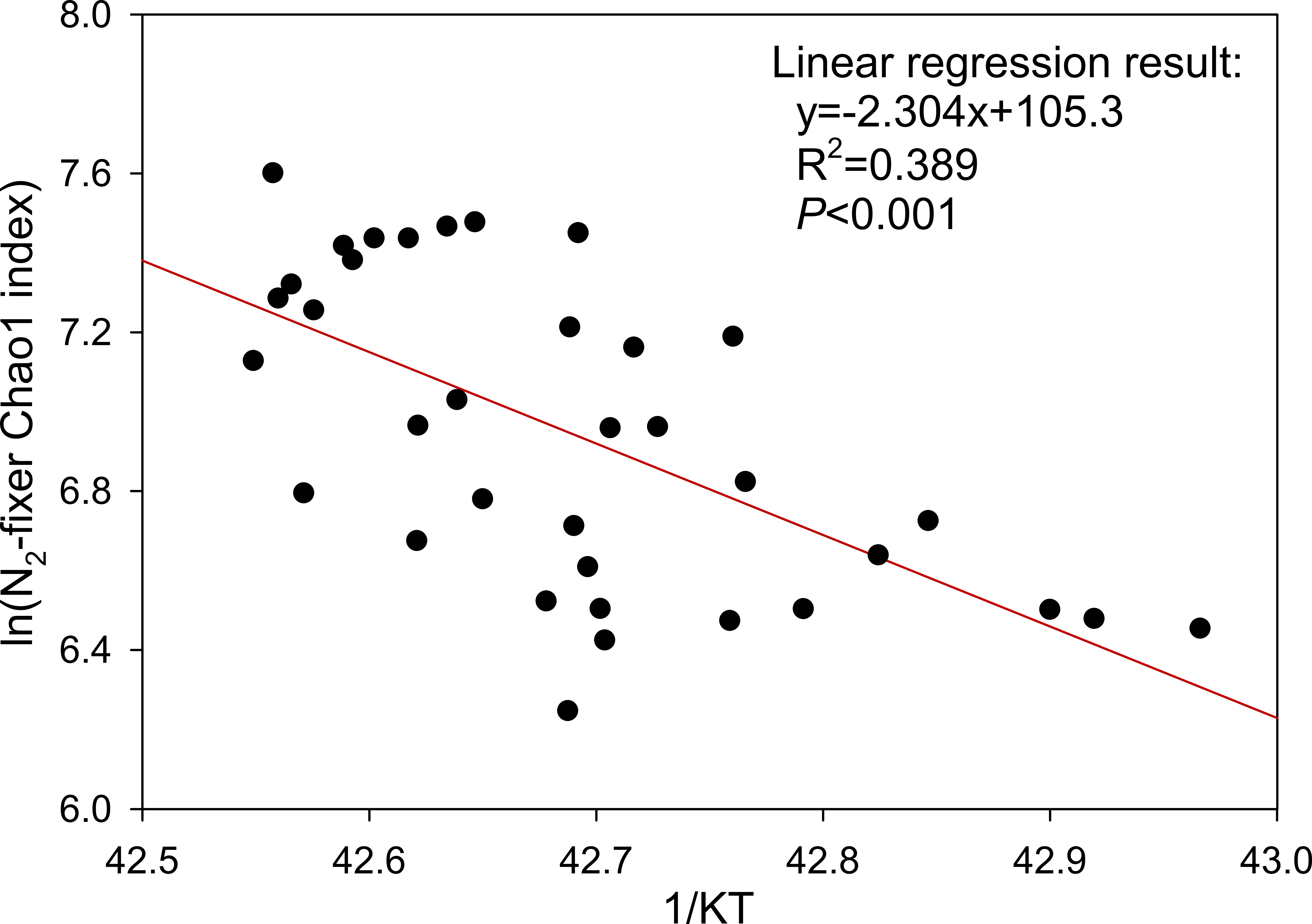

Supplement: FIG S5 [file mBio.02521-18-sf005.tif]
