# Supplementary material for: Long-Term Warming in Alaska Enlarges the Diazotrophic Community in Deep Soils
Source: mBio. 2019 Feb 26;10(1):e02521-18. doi: 10.1128/mBio.02521-18 (PMC6391920; doi:10.1128/mBio.02521-18)
Supplement: TABLE S2 [file mBio.02521-18-st002.docx]

**TABLE S2** Adonis tests of the effects of warming on diazotrophic community composition based from sequencing data and functional structure from GeoChip data for *nifH* genes*^a^*

| Layers | *nifH* sequencing*^b^* | | *nifH* GeoChip*^c^* | |
| --- | --- | --- | --- | --- |
|  | F | *P*-value | F | *P*-value |
| All*^d^* | 0.685 | 0.516 | 1.353 | 0.190 |
| L1 | 0.521 | 0.657 | 1.541 | 0.191 |
| L2 | 7.850 | **0.028***^e^* | 2.120 | ***0.057*** |
| L3 | 1.074 | 0.414 | 2.336 | **0.047** |
| L4 | 1.870 | 0.222 | 0.951 | 0.396 |

*^a^*Adonis, permutational Multivariate Analysis of Variance using distance matrices.

*^b^*Weighted Chao dissimilarity index is used for the dissimilarity analysis of diazotrophic communities based from sequencing data.

*^c^*Weighted Bray-Curtis dissimilarity index is used for the dissimilarity analysis of diazotrophic communities based on GeoChip data.

*^d^*All, all of 4 layers combined; L1, the upper organic layer; L2, the middle organic layer; L3, the lower organic layer; L4, the upper mineral layer.

*^e^*Significance: bold values, *P*≤0.05; bold and italic values, 0.05<*P<*0.1.
