# Supplementary material for: Long-Term Warming in Alaska Enlarges the Diazotrophic Community in Deep Soils
Source: mBio. 2019 Feb 26;10(1):e02521-18. doi: 10.1128/mBio.02521-18 (PMC6391920; doi:10.1128/mBio.02521-18)
Supplement: TABLE S3 [file mBio.02521-18-st003.docx]

**TABLE S3** α-diversity indices, within-treatment β-diversity, and relative abundances (sequence numbers) of *nifH* genes based on sequencing data*^a^*

| Indices/relative abundances | Layer | Control | Warming |
| --- | --- | --- | --- |
| Richness | L1*^b^* | 457.50^bc^ | 443.67^c^ |
|  | L2 | 599.17^bc^ | 817.50^a^ |
|  | L3 | 873.83^a^ | 845.17^a^ |
|  | L4 | 612.33^b^ | 456.67^bc^ |
| Chao1 index | L1 | 699.44^c^ | 658.86^c^ |
|  | L2 | 956.06^bc^ | 1413.27^a^ |
|  | L3 | 1494.71^a^ | 1481.36^a^ |
|  | L4 | 1037.62^b^ | 754.43^bc^ |
| Shannon index | L1 | 3.86^c^ | 3.88^c^ |
|  | L2 | 4.15^bc^ | 4.60^ab^ |
|  | L3 | 4.93^a^ | 4.88^a^ |
|  | L4 | 4.16^bc^ | 3.51^c^ |
| Within-treatment *nifH* β-diversity  (Bray-Curtis distance) | L1 | 0.591 | 0.589 |
|  | L2 | **0.44***^c^* | **0.648** |
|  | L3 | **0.548** | **0.606** |
|  | L4 | 0.576 | 0.659 |
| Within-treatment *nifH* β-diversity  (UniFrac distance) | L1 | 0.352 | 0.314 |
|  | L2 | **0.24** | **0.438** |
|  | L3 | **0.331** | **0.406** |
|  | L4 | 0.369 | 0.429 |
| Relative abundance of Alphaproteobacteria | L1 | 20420 | 24094 |
|  | L2 | 14873 | 16967 |
|  | L3 | 14263 | 15008 |
|  | L4 | **10682** | **7669** |
|  | Sum | 60238 | 63738 |
| Relative abundance of Betaproteobacteria | L1 | 8300 | 8302 |
|  | L2 | **17710** | **11768** |
|  | L3 | 11907 | 9935 |
|  | L4 | **4053** | **2745** |
|  | Sum | 41970 | 32750 |
| Relative abundance of Gammaproteobacteria | L1 | 5182 | 1507 |
|  | L2 | **1183** | **2716** |
|  | L3 | 4591 | 4254 |
|  | L4 | 16297 | 21230 |
|  | Sum | 27253 | 29707 |
| Relative abundance of Deltaproteobacteria | L1 | 439 | 339 |
|  | L2 | **1080** | **2018** |
|  | L3 | 1949 | 2253 |
|  | L4 | **2617** | **1623** |
|  | Sum | 6085 | 6233 |
| Relative abundance of Verrucomicrobia | L1 | **55** | **124** |
|  | L2 | **117** | **979** |
|  | L3 | **1426** | **2150** |
|  | L4 | **1098** | **1781** |
|  | Sum | 2696 | 5034 |
| Relative abundance of Cyanobacteria | L1 | 935 | 900 |
|  | L2 | 242 | 193 |
|  | L3 | 182 | 124 |
|  | L4 | **87** | **17** |
|  | Sum | 1446 | 1234 |
| Relative abundance of Firmicutes | L1 | 56 | 57 |
|  | L2 | **94** | **321** |
|  | L3 | 525 | 470 |
|  | L4 | 385 | 133 |
|  | Sum | 1060 | 981 |
| Relative abundance of Bacteroidetes | L1 | 10 | 67 |
|  | L2 | 78 | 145 |
|  | L3 | 464 | 600 |
|  | L4 | 75 | 55 |
|  | Sum | 627 | 867 |
| Relative abundance of Chlorobi | L1 | 1 | 23 |
|  | L2 | 7 | 268 |
|  | L3 | 13 | 521 |
|  | L4 | 13 | 18 |
|  | Sum | 34 | 830 |
| Relative abundance of Spirochaetes | L1 | 16 | 15 |
|  | L2 | **4** | **35** |
|  | L3 | 38 | 54 |
|  | L4 | 11 | 6 |
|  | Sum | 69 | 110 |
| Relative abundance of Actinobacteria | L1 | 10 | 0 |
|  | L2 | 33 | 1 |
|  | L3 | 26 | 8 |
|  | L4 | 71 | 8 |
|  | Sum | 140 | 17 |
| Relative abundance of Acidobacteria | L1 | 0 | 0 |
|  | L2 | **4** | **16** |
|  | L3 | 18 | 36 |
|  | L4 | 12 | 47 |
|  | Sum | 34 | 99 |
| Relative abundance of Nitrospirae | L1 | 1 | 0 |
|  | L2 | 0 | 3 |
|  | L3 | 2 | 5 |
|  | L4 | **24** | **94** |
|  | Sum | 27 | 102 |
| Relative abundance of Chloroflexi | L1 | 0 | 0 |
|  | L2 | 3 | 0 |
|  | L3 | 24 | 7 |
|  | L4 | 5 | 2 |
|  | Sum | 32 | 9 |
| Relative abundance of Epsilonproteobacteria | L1 | 5 | 2 |
|  | L2 | 2 | 0 |
|  | L3 | 0 | 1 |
|  | L4 | 0 | 2 |
|  | Sum | 7 | 5 |
| Relative abundance of Euryarchaeota | L1 | 0 | 0 |
|  | L2 | 0 | 0 |
|  | L3 | 2 | 4 |
|  | L4 | 0 | 0 |
|  | Sum | 2 | 4 |

*^a^*Lower case superscript letters that is not italic (e.g., ^a^, ^b^, ^c^, and ^ab^) in the table show the grouping result of ANOVA and LSD tests, verifying the significant differences among all values within each index. When the two numbers do not share the same lower-case letter, they are significantly different, and vice versa.

*^b^*Abbreviations: L1, the upper organic layer; L2, the middle organic layer; L3, the lower organic layer; L4, the upper mineral layer.

*^c^*Significance: bold values, *P*≤0.05 as determined by two-tailed *t*-test between warming and control.
