# Supplementary material for: Long-Term Warming in Alaska Enlarges the Diazotrophic Community in Deep Soils
Source: mBio. 2019 Feb 26;10(1):e02521-18. doi: 10.1128/mBio.02521-18 (PMC6391920; doi:10.1128/mBio.02521-18)
Supplement: TABLE S4 [file mBio.02521-18-st004.docx]

**TABLE S4** Pearson correlation of diazotrophic abundance between qPCR result and environmental variables

| R | All layers | L1*^a^* | L2 | L3 | L4 |
| --- | --- | --- | --- | --- | --- |
| Thaw duration (day per year) | -0.047 | 0.168 | ***0.571****^b^* | 0.041 | **0.677** |
| Moisture (VWC percentage within 0–20 cm) | **0.302** | -0.425 | 0.391 | 0.206 | **0.828** |
| Plant biomass (g/m^2^) | ***0.264*** | 0.349 | **0.678** | -0.074 | ***0.515*** |

*^a^*Abbreviations: L1, the upper organic layer; L2, the middle organic layer; L3, the lower organic layer; L4, the upper mineral layer.

*^b^*Significance: bold values, *P*≤0.05; bold and italic values, 0.05<*P<*0.1.
