# Supplementary material for: Long-Term Warming in Alaska Enlarges the Diazotrophic Community in Deep Soils
Source: mBio. 2019 Feb 26;10(1):e02521-18. doi: 10.1128/mBio.02521-18 (PMC6391920; doi:10.1128/mBio.02521-18)
Supplement: TABLE S5 [file mBio.02521-18-st005.docx]

**TABLE S5** Sequences, product sizes, and melting temperatures (T_m_) of qPCR primers of the top 11 abundant OTUs

| *nifH* OTU ID | Forward primer | Reverse primer | Product size/bp | T_m_/^o^C  (forward, reverse) |
| --- | --- | --- | --- | --- |
| OTU 109 | CGAGGACCTGGAACTCGATG | CCAACACGTCGTAGGACACA | 179 | 59.9, 59.97 |
| OTU 35 | GTTTCGGCGGTATCAAGTGC | ATCGTCATACGCGCCTTCTT | 116 | 59.9, 59.9 |
| OTU 7 | CGGCGTTATCACCTCGATCA | GTAGATTTCCTGCGCCTTGC | 142 | 59.97, 59.62 |
| OTU 67 | CGGAGAAGGGCACTATCGAG | CCGAGCACGTCATAGGAGAC | 196 | 59.69, 59.97 |
| OTU 54 | ATGAGAAGGCCCAGAACACG | GCCGAAGTCGACCTTCATCA | 95 | 60.04, 60.11 |
| OTU 188 | AGGGCATCAAGTGTGTCGAG | CGCCGAGCACATCATAGGAA | 141 | 60.04, 60.25 |
| OTU 277 | CTGGACGAGATCCTCAAGCC | AGCTGCTCCAGCATGTTGAT | 122 | 59.9, 60.03 |
| OTU 539 | GACGTGATGAAGGTCGGCTT | TCGAGGGAGTTGATCGAGGT | 110 | 60.39, 60.03 |
| OTU 86 | AGGCCCAGAACAGCATTCTC | GATGCGCTGATAGCCGTACT | 95 | 60.03, 60.04 |
| OTU 25 | CTGCACTCGAAGGCACAAAC | TCTTCGAGGAAGTTGACGGC | 188 | 60.04, 60.04 |
| OTU 262 | GGACCTGGAACTCGAAGACG | GCACGTCGTAGGAGACGTAG | 173 | 60.11, 59.97 |
